# Old trees are perceived as a valuable element of the municipal forest landscape

**DOI:** 10.7717/peerj.12700

**Published:** 2022-01-12

**Authors:** Marzena Suchocka, Magdalena Wojnowska-Heciak, Magdalena Błaszczyk, Agnieszka Gawłowska, Joanna Ciemniewska, Agata Jarska, Jakub Heciak, Beata Pachnowska

**Affiliations:** 1 Department of Landscape Architecture, Warsaw University of Life Sciences, Warsaw, Poland; 2 The National Institute of Telecommunications—the State Research Institute, Warsaw, Poland; 3 Warsaw, Poland; 4 Department of Civil Engineering and Architecture, Kielce University of Technology, Kielce, Poland; 5 Imas International sp. z o.o. Instytut badania rynku i opinii społecznej, Wrocław, Poland

**Keywords:** Municipal forest, Old hollow trees, Social survey, Forests’ visitors

## Abstract

Urban trees are important to maintain biodiversity and, therefore, need public acceptance. Few studies, however, have addressed the topic of social acceptability of old trees. The aim of this research was to examine city residents’ perception of old trees, including hollow-bearing ones, mainly in the aspect of safety and aesthetics. A total of 448 Warsaw municipal forest’ users expressed their opinions by completing an online questionnaire. Several methods were used to analyse the results of the study: the Chi-square test of independence, the Kruskal–Wallis H test, the Mann–Whitney U test and the Quartimax method of factor rotation analysis. The results revealed a correlation between the frequency of forest visits and the level of sensitivity toward old trees, which translates to less radical notion of danger and less radical decisions about cutting such trees down. Age of the respondents (56+) was a factor contributing to higher willingness to protect and care for old trees. The results also indicated that outdoor activity in the urban forest may increase ancient trees acceptance by developing emotional connection with them, and eventually contribute to their protection.

## Introduction

### Old trees

According to Zapponi et al. (2017), old trees present structural and functional characteristics fundamental for sustaining complex and unique assemblages of species. Old trees are an invaluable element of natural environment (Alexander, 2001; Butler, Rose & Green, 2001; Le Roux et al., 2015) as they bring substantial benefits to birds (Ferenc, Sedláček & Fuchs, 2014), mammals (*e.g.*, red squirrel), bryophytes, lichens, and fungi (Birch et al., 2021), saproxylic insects (Siitonen & Ranius, 2015), edge-space bats (Polyakov, Weller & Tietje, 2019), and ensure ecosystem diversity in often hard to reach regions (Hall & Bunce, 2011). Veteran and dead trees are recognized as Pan-European indicators of sustainable management (Radu, 2006).

Although old trees sometimes grow in marginal habitats (Orłowski & Nowak, 2007), they are an indispensable element of cultural landscape (Blicharska & Mikusiński, 2014; Benner, Nielsen & Lertzman, 2021). Trees play a part in intangible heritage. People value trees as emblems, symbols, or gifts from Gods (Roudavski & Rutten, 2020). In general they are associated with social cohesion (Holtan, Dieterlen & Sullivan, 2014), contribute to urban neighbourhoods’ aesthetic quality and enhance human mental and physical health and well-being (De Vries et al., 2003; Hansmann, Hug & Seeland, 2007; Berman, Jonides & Kaplan, 2008; Maas et al., 2009; Donovan et al., 2011; Korpela et al., 2014; Tyrväinen et al., 2014; Taylor, Gartner & Morrell, 2002).

With age, hollows and cavities appear in a tree, which may affect the perception of such an old tree but also certain decisions concerning its conservation or felling. Hollows are found in old trees, whether living or not. A tree hollow, usually in the form of a semi-enclosed cavity, forms naturally in the trunk or branch of a tree (Gilman, 2020). Hollows may form as the result of physiological stress from natural forces. The size of the cavity may be related to the age of the tree (Gibbons & Lindenmayer, 2002). Tree hollows take on different shapes: an elongated slot, a chamber with a round or oval entrance hole, an irregular hole. In forestry, a cavity is problematic as it is considered to weaken the tree (Mattheck, Bethge & Tesari, 2006; Fink, 2009), especially in mechanical terms, where the size of hollows is assumed to indicate the weakening of stem strength. This point of view, however, is being questioned given the anisotropic architecture of the tissues (Spatz & Niklas, 2013). In general, the tree hollows are rare in urban space and valuable for wildlife, therefore is it crucial to protect them (Stojanovic et al., 2014; Rueegger, 2017; Edworthy et al., 2018). According to Janzen, (1976), a rotten core is often an adaptive trait. It is a site of animal nests and microbial metabolism that may result in the steady fertilization of soil under the tree. For this reason, hollow-bearing trees are expected more frequently in nutrient-poor sites (Janzen, 1976; Jim, 1998). Moreover, as reported by Tailor (2002), heartwood plays no role in nutrient storage or water transport and offers no structural advantage over sapwood. It is metabolically inactive, meaning that chemical defences cannot be replenished when they decay (Ruxton, 2014). In cities, tree cavities suitable for nesting are less common than in other areas, Thus, enhancing biodiversity and resilience of urban areas to conserve the tree cavity resource across the landscape (Stojanovic et al., 2014; Rueegger, 2017; Edworthy et al., 2018) is essential.

In addition to their uniqueness, old trees are especially susceptible to removal in urban landscapes (Jim, 1998; Terho, 2009; Nagendra & Gopal, 2010). This is due to the potential safety risks posed to the public and infrastructure by falling branches or up-rooted or windfallen trees (Sterken, 2006; Cockle, Martin & Wesołowski, 2011; Manning et al., 2013). People are usually concerned about any visible signs of damage. While a hollow-bearing tree might appear unstable, it is rarely a cause for concern—in fact, many such trees have stood and thrived for hundreds of years and can still appear stable and healthy (Prince Tree Surgery, 2020b; Loeb, 2011; Rhoades, 2020; Williams, 2015; Anonymous 2020d). Unfortunately, not the research-based evidence or expert recommendation but people’s subjective perception plays a decisive role when it comes to taking a decision about the removal of old, probably hollow trees from the garden, neighbourhood street or even tourist trails in municipal forests (Suchocka, Błaszczyk & Kosno, 2019; Suchocka & Kimic, 2019). It is thus advisable to study people’s knowledge, attitudes and emotions connected with old trees and certain characteristics related to the tree’s senior age such as hollows, cavities, fungi on the trunks or branches, *etc.*

### Trees in social studies

Social acceptability is one of the criteria guiding urban forest management decisions (Brunson et al., 1996). The most common components of the respondent profile that significantly contribute to the variations in the research results concerning tree perception or general approach to nature conservation are the education level (Camacho-Cervantes et al., 2014; Kangur, 2015), the age of the respondents (Koyata et al., 2021; Wojnowska-Heciak et al., 2020), and the place of residence (Oyebade BA & Itam, 2013; Suchocka, Jankowski & Błaszczyk, 2019). People from more urbanized areas are more appreciative of trees than rural dwellers (Oyebade BA & Itam, 2013). With age, the need for having trees in the surrounding is more visible (Wojnowska-Heciak et al., 2020), and with a higher level of education, materialist values (focused on physical security and economic well-being) change gradually toward post-materialist values (focused on life quality), acceptance of biodiversity, including presence of mature trees in urban forests (Kangur, 2015; Hauru et al., 2014; Gerstenberg & Hofmann, 2016; Douglas, Lennon & Scott, 2017) and acknowledging the role of trees in reducing dust, smog, and noise (Nowak, Crane & Stevens, 2006).

A series of studies found that the main motivation for tree planting or conservation in urban landscapes focuses on aesthetics (Summit & McPherson, 1998; Kirkpatrick, Davison & Daniels, 2012; Conway, 2016) as the most common positive attribute residents associate with trees (Flannigan, 2005; Pataki et al., 2013; Camacho-Cervantes et al., 2014; Avolio et al., 2015). People appreciate terrestrial colours that nature brings to the urban context (Doherty, 2011) and its positive impact on the mood, like in the case of prospective passengers waiting at transit stations, where the presence of mature trees reduces the perceived waiting time (Sommer & Summit, 1995).

But still, the perception and attitudes towards trees (in particular, mature trees) have not been thoroughly investigated (Kirkpatrick, Davison & Daniels, 2013). The reasons for tree removal most often reported in the literature are often related to diseases or advanced age, followed by problems they cause (*e.g.*, structural damage caused by roots) (Summit & McPherson, 1998; Kirkpatrick, Davison & Daniels, 2012). However, there are also many healthy tree removals that occur due to insufficient knowledge of planters (Conway, 2016). Although people are able to recognize the differences between a dead tree and a living tree, and are aware of the risks connected with the collapse of unhealthy trees, they present ambivalent approach to ultimate solutions (Nali & Lorenzini, 2009). People accept maturity in trees to a certain extent. Decaying logs and dead wood are not accepted, disliked by the public, and recognized as a reduction of scenic beauty and recreational values of forests (Brown and Daniel, 1984; Ribe, 1999; Liao & Nogami, 1999; Tyrväinen, Silvennoinen & Kolehmainen, 2003; Ribe Robert, 2009; Edwards et al., 2012). This general perception changes when the role of old trees in the natural environment is properly understood by urban forest visitors (Hauru et al., 2014; Gundersen et al., 2016).

### Purpose and scope of the study

Biological diversity of Polish urban forests associated with the presence of old, including hollow trees is protected today by various traditional forms of nature conservation (Ustawa 1991/ Act 1991, 1991; Ustawa 2004/Act 2004, 2004; Ustawa 2014/Act 2014, 2014) and pro-ecological, sustainable forest management (Referowska-Chodak, 2010). However, the retention of ancient urban trees is problematic because of public pressure. The presence of old urban trees is important to maintain biodiversity, as well as urban ecosystem health/ stability but setting the safety limit raises most controversy and disagreement. The issue of old trees does not appear directly in international or national laws related to nature protection and forestry. Instead, it is present in all current industry guidelines for forest management, certification systems and scientific publications (Referowska-Chodak, 2010) and could be adapted in urban landscape with public acceptance. It is crucial to study how city residents and especially urban forests visitors perceive old (*Ancient* or *Senescence* stages) hollow-bearing trees. Rarely undertaken, such a study could directly influence city policies towards urban tree management. It is worth pointing out that Warsaw is one of the two EU cities with natural forests within city limits. We studied the perception of old trees among people visiting these forests.

Based on the literature review and our own study, we aimed at understanding how city residents perceive old trees, whether they feel that the trees pose a risk to them or whether they think old trees are valuable and aesthetically pleasing. The main hypothesis is that old trees are accepted and people who often visit forests feel more related to nature (Haskell, 2012; Häggström, 2019) and perceive old trees more positively than those who are not keen forest-goers.

## Materials and Methods

### Study area—municipal forests in Warsaw

**Figure 1:**
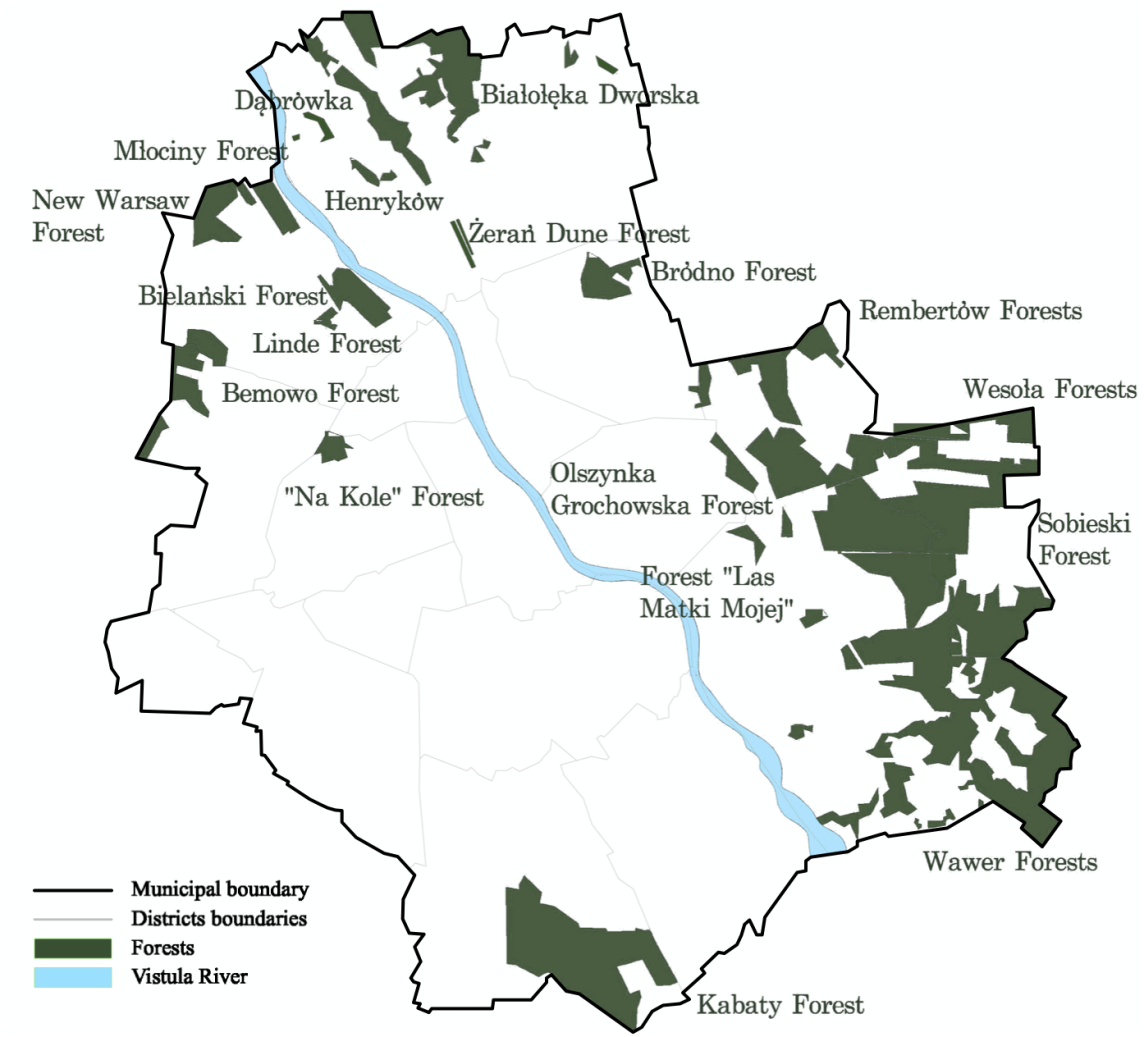
Location of municipal forests in Warsaw.

Municipal forests in Warsaw are the main living environment of old trees. The definition of municipal forest in Poland differs from the definition used worldwide. According to the Ministry of the Environment, “the forest is a compact area of at least 0.10 ha, covered with forest vegetation (forest crops) - trees and shrubs and forest undergrowth or temporarily deprived of it”. Besides, to be able to talk about the forest, the area must be designated for forest production, be a nature reserve or be part of a national park or be entered in the register of monuments (Ministry of the Environment of Poland, 2020a). Warsaw is one of few European capitals in which forests are located within the city limits and occupy large areas. The current forest cover in Warsaw is 15% of its area (Fig. 1). Private forests, under the supervision of the Mayor of the Capital City of Warsaw, occupy about 3,200 ha, while forests owned by the State Treasury under the management of the State Forests cover an area of about 1,300 ha (Piekielniak, 2018; National Forests of Poland, 2020c).

### Social study

An online survey using Google Forms was carried out in the period between 14 and 25 January 2020. The aim was to check the attitudes towards old trees among municipal forests’ users and their approach to the health management methods applied. The questionnaire was developed based on expert knowledge. There was no pilot study, but the survey questions were consulted with the academics and students from the Warsaw University of Life Sciences (SGGW).

The questionnaire consisted of four sections, including respondents’ general metrics, the key question whether the respondent visits municipal forests or not, questions about activities taken in municipal forests, and statements concerning attitudes towards old trees. Participation was anonymous and voluntary. At each stage, the respondents could quit the survey without completing it. The survey was addressed to people who visit municipal forests in Warsaw and live in Warsaw or in close vicinity (Bieńkowska et al., 2015). In Warsaw, the survey was distributed among social media discussion group members from Bielany district (because of the close vicinity of the biggest municipal forests: Mlocinski and Bielański Forests) and Ursynów district (because of close vicinity to the Kabacki forest). Other participants included individuals that felt close to the nature of Warsaw and surrounding area and a group of Mazovian mushroom pickers who visited the forests in Warsaw at least once in the past year (Fig. 1). A total of 526 respondents were recruited. The majority (85.3%) of the people who received the questionnaire visited Warsaw forests. People who do not visit forests did not continue the survey. Ultimately, 448 people completed the questionnaire. Several methods were used to analyse the results of the study: the *χ*2 test (Corder, 2009), the Kruskal–Wallis H test (the non-parametric equivalent of analysis of variance (ANOVA)) (Kruskal & Wallis, 1952), the Mann–Whitney U test and the Quartimax method of factor rotation analysis (Mulaik, 2009). Quatrimax allowed to bring out a clearer picture of the factors and gave the most readable results. We checked other rotations as well, the results were similar, although not so clear. In the case of Quartimax, the rotation criterion is to maximize the variance of the squared factor loadings for each variable, given the number of factors, the given resources of common variation, and the orthogonality of the factors is maintained. It is a special case of orthomax rotation, which maximizes the sums of squares of the coefficients across the resultant vectors for each of the original variables, as opposed to varimax, which maximizes the sums of squares of the coefficients within each of the resultant vectors (Jackson, 2005). The Mann–Whitney U test is a special case of the Proportional odds model, allowing for covariate-adjustment. The Mann–Whitney U test is preferable to the *t*-test when the data are ordinal but not interval scaled, in which case the spacing between adjacent values of the scale cannot be assumed to be constant. As it compares the sums of ranks, the Mann–Whitney U test is less likely than the *t*-test to spuriously indicate significance because of the presence of outliers. However, the Mann–Whitney U test may have worse type I error control when data are both heteroscedastic and non-normal. But our data was neither heteroscedastic (ie groups differed in response variation), nor non-normal. Therefore, for both tests (the Kruskal–Wallis H test and the Man-Whitney U test) we did not use any correction tools, including the Bonferroni corrections. We used the Bonferroni correction for Chi-2 test.

The data analysis was performed using the SPSS Statistics (SPSS) (Version 25) (Fox, 2005).

Among the respondents, 321 women (71.7%) and 127 men (28.3%) took part in the study. The least numerous group comprised people over 56 years of age. Responses from the residents of the city with a population exceeding 500,000 amounted to 75.2%. The survey was also completed by 68 rural residents, which constituted 15.2% of votes. Among the respondents there were also residents of town with less than 25,000 inhabitants (12 responses - 2.7%), between 26,000 and 100,000 inhabitants (21 responses - 4.7%) and between 101,000 and 500,000 inhabitants (10 responses –2.2%). Most respondents declared completing higher education—as much as 75.2%. Percent of persons who completed the secondary level of education and students was 12.5% and 12.3%, respectively (Table 1).

**Table 1:** The respondents’ structure in terms of demographic characteristics (gender, age, size of place of residence, and education.

		**Number of respondents**	**% from N in the column**
Gender	Female	321	71.7%
	Male	127	28.3%
Age	18–25	102	22.8%
	26–35	119	26.6%
	36–45	115	25.7%
	46–55	70	15.6%
	>56	42	9.4%
Place of residence	Village	68	15.2%
	Town 25,000 residents	12	2.7%
	Town 25–100,000 residents	21	4.7%
	City 101–500,000 residents	10	2.2%
	A city with a population of over 500,000 residents	337	75.2%
Education	Elementary	7	1.6%
	Vocational	5	1.1%
	Secondary	56	12.5%
	Students	55	12.3%
	Higher	325	72.5%

## Results

### Frequency of forest visits

**Figure 2:**
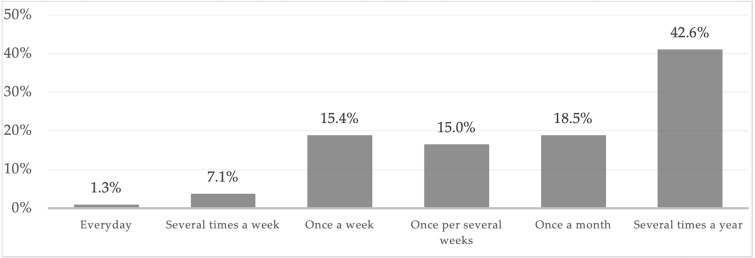
Distribution of answers to the question “How often do you visit municipal forests in Warsaw?”

Over half of the respondents visit Warsaw’s municipal forests at least once a month. Generally, the forests are visited once a week or less often. Only 8.4% of the respondents go the forest several times a week or every day (Fig. 2).

The most appreciated aspect of municipal forests is the possibility of contact with nature as indicated by 91.7% of the respondents. Two thirds of the survey participants like forests because of the beautiful scenery (65.2%), and slightly less for comfortable hiking trails (56.7%). Respondents visit municipal forests also because of the possibility of doing sports (26.1%) and forest infrastructure—picnic sites (22.3%) and playgrounds (9.6%).

### Beliefs concerning old trees

Factor analysis with the use of the Quartimax method showed the occurrence of three factors (Tables 2–3):

**Table 2:** Rotated components matrix including factor loadings.

	Component		
	1 W	2 S	3 CP
*Old trees increase the attractiveness of municipal forests.*	**.745**	**−.160**	**.035**
*Old trees are more valuable to the environment than younger trees.*	**−.243**	**.701**	**−.134**
*It is better to replace an old tree with a younger one.*	**.658**	**−.314**	**.026**
*Fruiting bodies of fungi on the trunk or branches do not mean a death sentence for the tree on which they grow.*	**−.065**	**.452**	**−.201**
*Hollow trees pose a greater threat than trees without hollows.*	**.642**	**.154**	**−.102**
*Old decaying interior of trees must be cut out immediately.*	**.667**	**−.141**	**.327**
*Old trees increase the attractiveness of municipal forests.*	**−.299**	**.596**	**.024**
*Dying trees should be cut down even if they are natural monuments.*	**.587**	**−.066**	**.279**
*Trees in municipal forests should undergo the same care as street trees.*	**.150**	**−.076**	**.795**
*Cutting branches improves the health of even old trees.*	**.238**	**−.073**	**.782**
*There are many methods to determine the actual health of very old trees.*	**.087**	**.594**	**.122**

**Notes.**

Method of extracting factors - main components.

Rotation method - Quartimax with Kaiser normalization.

**Table 3:** Matrix of transformed components.

Component	1 W	2 S	3 CP
1	**.797**	**−.435**	**.419**
2	**.116**	**.791**	**.600**
3	**.593**	**.430**	**−.681**

**Notes.**

Method of extracting factors - main components. Rotation method - Quartimax with Kaiser normalization.

1. Non-acceptance of old trees (1W)
2. Acceptance of old trees and biodiversity (2S)
3. Approach to cutting trees (3CP).

**Figure 3:**
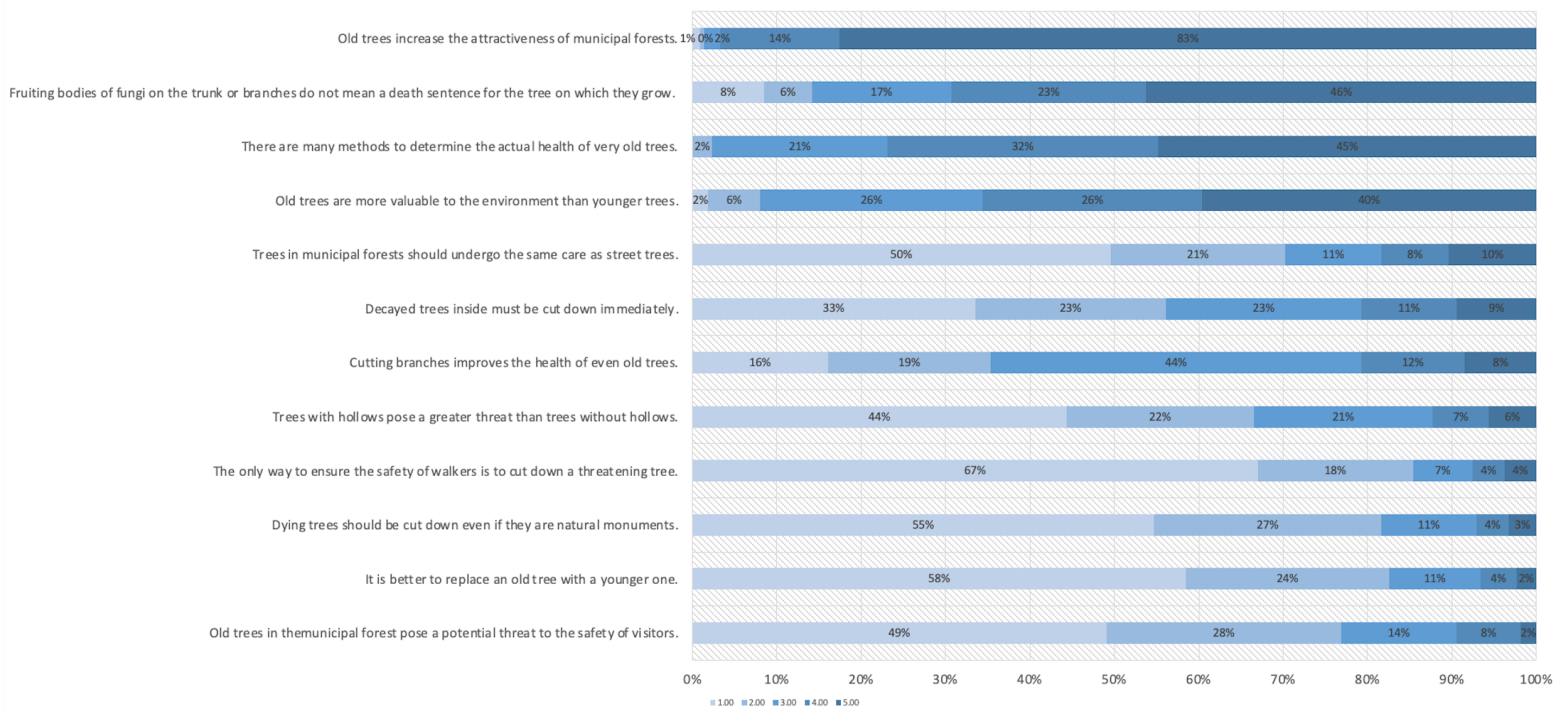
Distribution answers on of a scale of 1–5 to questions related to tree care. Key: 1.00 strongly disagree, 2.00 disagree, 3.00 I don’t know, 4.00 I agree, 5.00 I strongly agree.).

**Figure 4:**
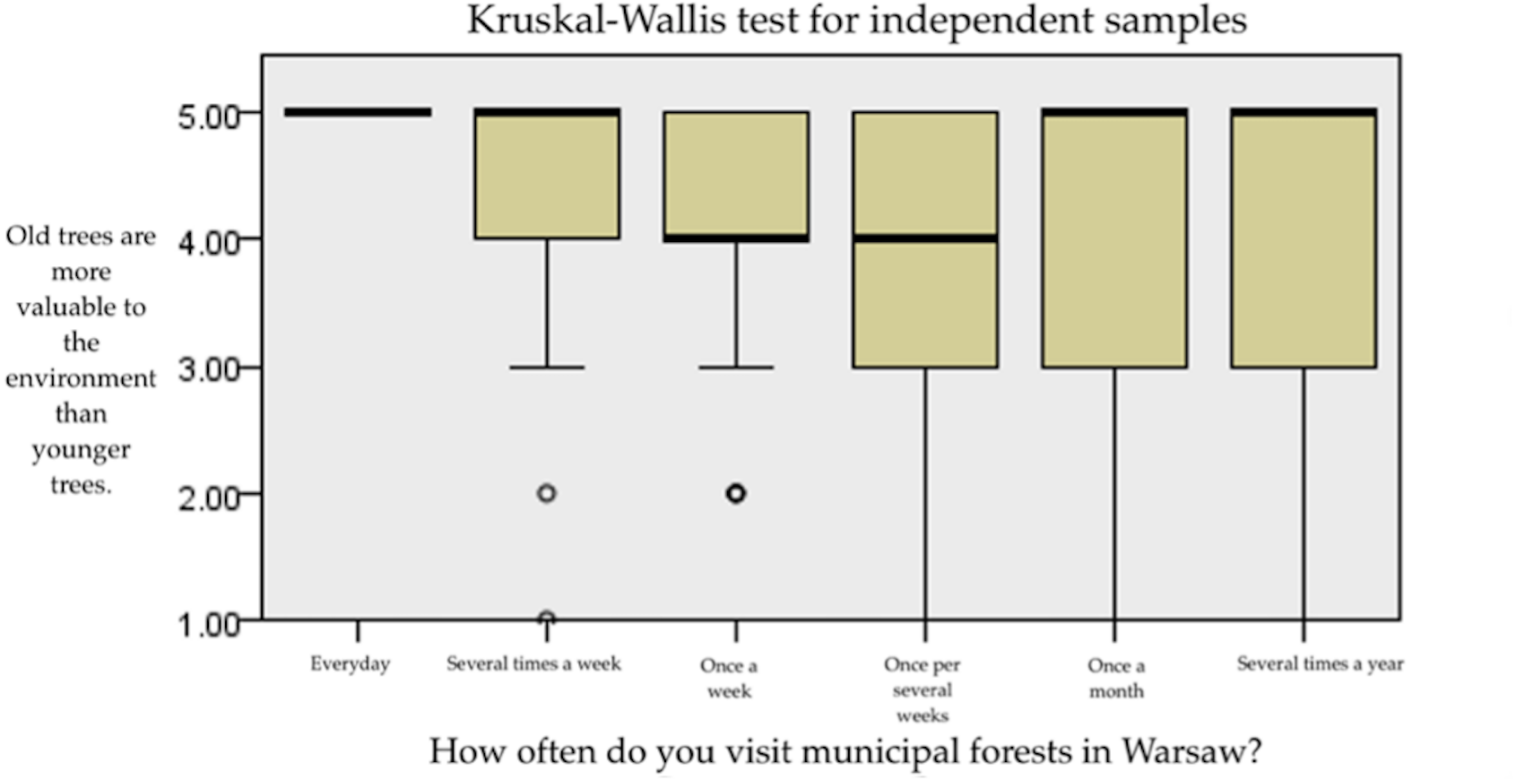
Box-plot of the answer to the question “Old trees are more valuable element of the environment than the young trees”.

Analysis of each statement from Fig. 3 (1W, 2S, 3CP) shows that as for old tree characteristics, there is a visible advantage of responses confirming the acceptance of old trees, including hollow/decayed trees, over the responses indicating non-acceptance in the whole sample. Considering only the statements saturated with the acceptance factor (2S, Fig. 3), a very positive attitude towards the presence of old trees, including those with hollows, and their role as an attractive and valuable landscape element, is clearly visible. People, in general, are not afraid of old trees and do not consider them threatening. Both statements referring to the 1W factor (Fig. 4) obtained a significant advantage indicating disagreement to felling such trees. People do not want to replace old trees with new ones. Only 6% of respondents agreed with the statement that old trees should be replaced with young ones. The majority of people believe that old trees increase the attractiveness of municipal forests (97%), slightly fewer believe that *old trees have more environmental value than young trees* (66%). Over three quarters of respondents are convinced that *there are many methods to determine the actual health condition of very old trees* (72%), and two thirds that *fruiting bodies of fungi on a trunk or branches are not a death sentence for the tree on which they grow* (69%). The statement that showed the respondents’ unawareness was *cutting branches improves the health of even old trees* - 44% of people were unable to determine whether this was true.

More than three quarters of the respondents disagree with statements *the only way to ensure the safety of walkers is to cut down a threatening tree* (85%), *replace the old tree with a younger one* (82%), *dying trees should be cut down even if they are natural monuments* (82%) *whether old trees in the city forest pose a potential threat to the safety of visitors* (77%). More than two-thirds of people do not think that *trees in forests should receive the same care as trees on the streets* (71%) and *hollow trees pose a greater threat than trees without hollows (* 66%). More than half disagree with the statement that *old* decaying interior of trees *must be cut out immediately* (56%) (Fig. 3). The respondents are quite sceptical about radical behaviour towards old trees. As a result of the confirmatory factor analysis, the first hypothesis claiming that old including hollow trees are accepted was partially confirmed (Fig. 3). Old trees are accepted in urban forests but to a certain extent, as long as they are perceived as aesthetically pleasing and posing no danger.

The table below shows that age and education level are important for the acceptance of old trees. The youngest respondents are against old trees in most cases. This is partly related to the level of education, which was not high among people aged 18-25 years. Therefore, the first hypothesis is supported by the results, but not by the entire sample (Table 4). Respondents coming from the village differ in the attitude towards danger posed by old trees (factor 1W, Fig. 3) from the residents of the city with a population exceeding 500,000. Village inhabitants are less afraid of old trees than city residents, what partly supports the second hypothesis assuming, that people who visit forests more often perceive old trees more positively, the fears of the old trees decline.

**Table 4:** Results of factor analysis in terms of demographic characteristics (1 W - Non-acceptance of old trees; 2 S - Acceptance of old trees and biodiversity, 3 CP - Approach to cutting trees).

	In all	Gender		Age					Place of residence					Education					How often do you visit municipal forests in Warsaw?		
	In all	Female	Male	18–25	26–35	36–45	46–55	>56	Village	Town 25,000 residents	Town 25–100,000 residents	City 101 –500,000 residents	A city with a population of over 500,000 residents	Elementary	Vocational	Secondary	Students	Higher	Once a week or more often		Several Times a week/ Once a month	Several times a year
	(A)	(A)	(B)	(A)	(B)	(C)	(D)	(E)	(A)	(B)	(C)	(D)	(E)	(A)	(B)	(C)	(D)	(E)	(A)		(B)	(C)
1W	*1,9*	*1,9*	*2,0*	*2,2*	*2,0*	*1,8*	*1,7*	*1,8*	*2,1*	*2,0*	*1,9*	*1,9*	*1,9*	*2,1*	*2,0*	*1,9*	*2,1*	*1,9*	*1,8*		*2,0*	*1,9*
2S	*4,3*	*4,3*	*4,2*	*4,2*	*4,3*	*4,3*	*4,4*	*4,5*	*4,3*	*4,3*	*4,3*	*4,1*	*4,3*	*3,9*	*4,6*	*4,2*	*4,2*	*4,3*	*4,3*		*4,3*	*4,3*
3CP	*2,4*	*2,5*	*2,4*	*2,5*	*2,5*	*2,3*	*2,2*	*2,6*	*2,6*	*2,9*	*2,5*	*2,1*	*2,4*	*3,1*	*3,2*	*2,8*	*2,5*	*2,3*	*2,4*		*2,6*	*2,3*

	In all	Gender		Age						Place of residence				Education					How often do you visit municipal forests in Warsaw?		
	In all	Female	Male	18–25	26–35	36–45	46–55	>56	Village	Town 25,000 residents	Town 25–100,000 residents	City 101 –500,000 residents	A city with a population of over 500,000 residents	Elementary	Vocational	Secondary	Students	Higher	Once a week or more often	Several Times a week/ Once a month	Several times a year
	(A)	(A)	(B)		(A)	(B)	(C)	(D)	(E)	(A)	(B)	(C)	(D)	(E)		(A)	(B)	(C)	(D)	(E)	(A)		(B)	(C)
1W				*B C D E*	*D*				*E*										
2S		*B*				*A*	*A B*	*A B*							*A*			*A*	
3CP														*E*		*E*			

**Notes.**

Results are based on two-tailed tests, assuming equal variance, with a significance level of 0.05. For each significant pair, the smaller category appears below the category with the larger mean.

### Beliefs concerning old trees versus the frequency of forest visit and nature/infrastructure preferences

To verify the second hypothesis whether beliefs related to trees depend on the frequency and purpose of visits to the forest, several analyses were carried out. The basis of the first analysis was the frequency of visits in municipal forests. Groups of people visiting forests with different frequencies are not equal (*χ*2 = 270.93; *p* < 0.001), therefore the Kruskal–Wallis H test (the non-parametric equivalent of ANOVA) and the Mann–Whitney U tests was chosen. It was assumed that the Likert scale used in the survey can be treated as a quantitative scale. Definitely the Kruskal–Wallis H test shows that the answers between the groups differ (Fig. 4). We have performed additional Mann–Whitney U tests, used as a post-hoc, to determine differences in more detail (Table 5). The analysis using the Kruskal–Wallis H test, and Mann–Whitney U test (Table 5) used as a post hoc test, revealed that people visiting forests every day significantly more often agree with the statement that old trees are more valuable for the environment than younger trees. Post-hoc analysis showed that people who go to the forest every day have a significantly different opinion of “Old trees are more valuable element of the environment than the young trees” from people who go to the forest once a week, once a few weeks, once a month and several times a year. Additionally, you can see differences in views between people who walk once a few weeks and several times a year.

**Table 5:** Frequency of forest visits and answers to the question “Old trees are more valuable element of the environment than young trees”.

		Old trees are more valuable element of the environment than the young trees	
Pairwise comparisons		**U Manna–Whitney test**	**Statistical significance**
Everyday	Several times a week	54	0.051
Everyday	Once a week	102	0.024
Everyday	Once per several weeks	78	0.009
Everyday	Once a month	129	0.030
Everyday	Several times per year	291	0.025
Several times a week	Once a weeks	1044.5	0.636
Several times a week	Once per several weeks	850.5	0.079
Several times a week	Once a month	1266	0.671
Several times a week	Several times per year	2887	0.586
Once a week	Once per several weeks	1924	0.073
Once a week	Once a month	2850	0.957
Once a week	Several times per year	6559.5	0.952
Once per several weeks	Once a month	2316	0.061
Once per several weeks	Several times per year	5381.5	0.038
Once a month	Several times per year	7853	0.894

**Figure 5:**
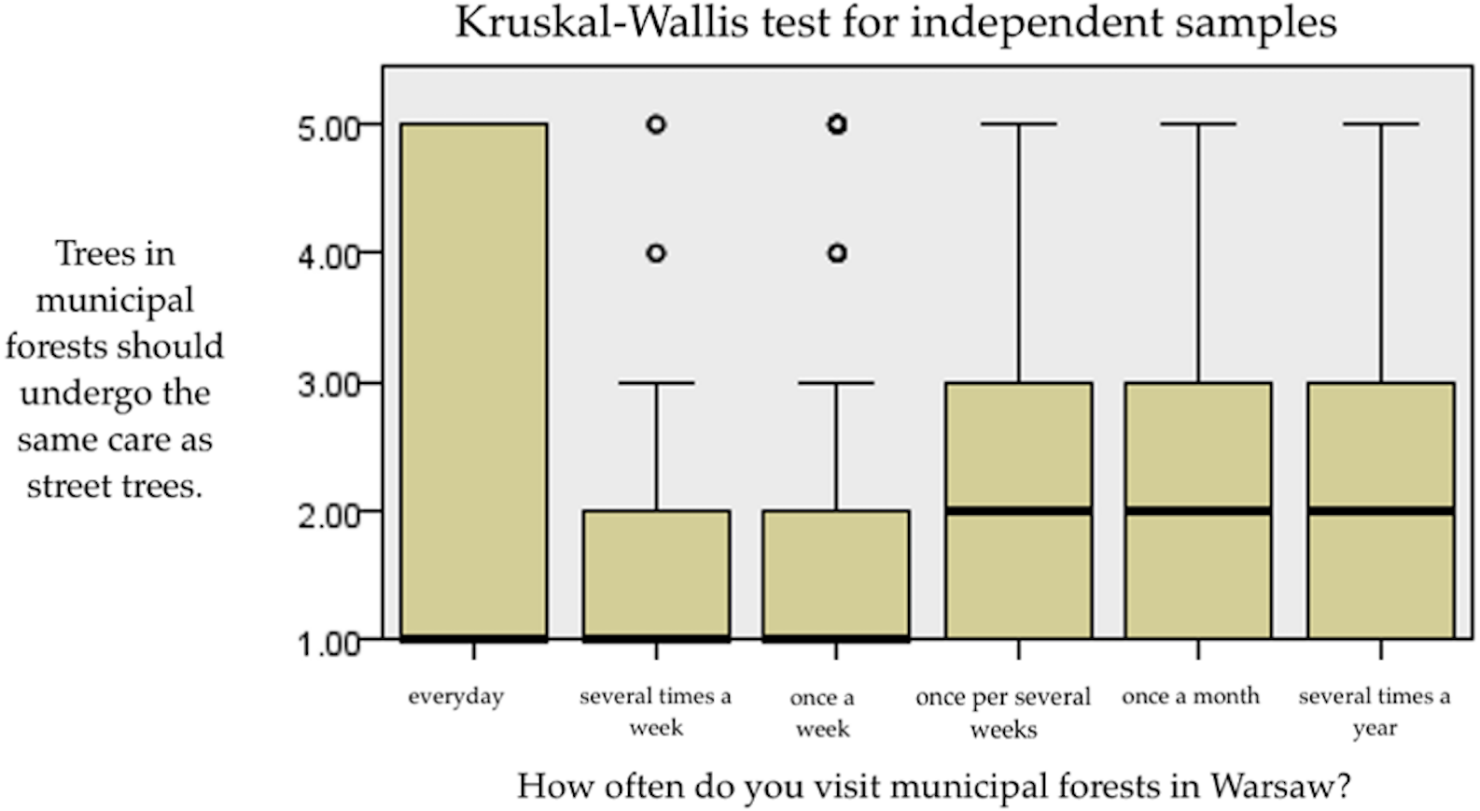
Box-plot of the answer to the question “Trees in municipal forests should receive the same care as street trees”.

The analysis with the Kruskal–Wallis H test showed differences between people visiting forests several times a week and people who visit the forest once a month (Fig. 5) in terms of care for municipal forest trees and street trees. People visiting forests several times a week significantly less often agree from people once a few weeks, once a month, people once a week from people once a month, people once a month from people several times a year with the statement that trees in reserves should receive the same care treatments as trees on the streets. The analysis illustrates that people who have more frequent contact with urban forests are more likely not to take radical decisions about tree treatment (*e.g.*, cutting) (Table 6).

**Table 6:** Frequency of forest visits and answers to the question “Trees in municipal forests should receive the same care as street trees”.

		Trees in municipal forests should receive the same care as street trees	
Pairwise comparisons		**U Manna–Whitney test**	**Statistical significance**
Everyday	Several times a week	86	0.627
Everyday	Once a week	205	0.965
Everyday	Once per several weeks	180	0.657
Everyday	Once a month	214	0.552
Everyday	Several times per year	547	0.840
Several times a week	Once a weeks	984.5	0.321
Several times a week	Once per several weeks	792	0.024
Several times a week	Once a month	895	0.004
Several times a week	Several times per year	2475.5	0.064
Once a week	Once per several weeks	1940	0.083
Once a week	Once a month	2199.5	0.009
Once a week	Several times per year	6020.5	0.252
Once per several weeks	Once a month	2553.5	0.372
Once per several weeks	Several times per year	5925	0.339
Once a month	Several times per year	6719.5	0.035

The comparison of the opinions in terms of gender or education performed using the Chi-square test did not show such significant differences as in the case of the age of the respondents. People aged 18–35 would be more likely to make radical decisions about cutting down old and hollow trees than people aged 56+. Another correlation was noticed while analysing the frequency of forest visits (Table 7). People who go to the forest once a week would be much more likely to decide to remove a tree than those who visit a few times a week. People who disagree with the statement *the only way to ensure the safety of walkers is to cut down a threatening tree* declared visiting the forest a few times a week more often than those visiting a few times a year (Table 7).

**Table 7:** Results of chi-square test for demographic characteristics (gender, age, frequency of park visits).

Gender		Age					How often do you visit municipal forests?					
		Female	Male	18–25	26–35	36–45	46–55	>56	Everyday	Several times per week	Once a week	Once per several weeks	Once a month	Several times per year
		(A)	(B)	(A)	(B)	(C)	(D)	(E)	(A)	(B)	(C)	(D)	(E)	(F)
Chi2 test		*χ*2=0.09; *p*= not significant		*χ*2=18.17; *p* <0,05					*χ*2=20.38; *p* <0,05					
The only way to ensure the safety of walkers is to cut down a threatening tree	Yes	45.8%	47.2%	55.9% D	52.1% D	44.3%	28.6%	40.5%	0.00%	25.0%	55.1% B	47.8%	44.6%	48.2%
	No	50.8%	49.6%	40.2%	44.5%	51.3%	68.6% A B	59.5%	100.00%	75.0% C F	40.6%	52.2%	50.6%	47.6%
	I don’t know	3.4%	3.1%	3.9%	3.4%	4.3%	2.9%	0.0%^1^	0.00%	0.0%^1^	4.3%	0.0%^1^	4.8%	4.2%

**Notes.**

“The results are based on two-tailed tests. For each significant pair, the category with the smaller column proportion appears under the category with the larger column proportion. Significance level for capital letters (A, B, C): .052”.

This category was not used in the comparisons because its column proportion is zero or one.

Tests are adjusted for all pairwise comparisons within each inner subtable using the Bonferroni correction.

Depending on the variable–the letters are conventional. The letter always refers to the column heading, *i.e.* A–age 18–25. A–female, A–primary education.

Considering the declaration what were the elements most appreciated by the respondents (Fig. 3), we distinguished two groups of visitors. The first group covered people who value forest for the contact with nature and beautiful landscapes, and the second group that pointed good quality infrastructure and opportunity to do different activities there. As question illustrated in Fig. 3 was a multiple-answer question, the division was not exclusive. Therefore, we had 425 respondents who declared that they prefer nature and 293 who declared to prefer infrastructure. Statistically significant differentiation is noticeable in four areas (Table 8). People who appreciate nature, value old trees, perceive them as a valuable element of the landscape. People who value infrastructure more than nature in the forest are not willing to observe the occurrence of decaying or dying trees (Table 8).

**Table 8:** Factors’ mean values in relation to what people appreciate more in the forest.

	In municipal forests, you appreciate the most		
	Those who appreciate nature more	Those who appreciate infrastructure more	All
	Mean	Mean	Mean
Non-acceptance of old trees (1W)	**1.9**	**2.0**	**1.9**
Acceptance of old trees and biodiversity (2S)	**4.3**	**4.3**	**4.3**
Approach to cutting trees (3CP)	**2.4**	**2.4**	**2.4**

**Comparisons of means in columns**			
	In municipal forests, you appreciate the most		
	Those who appreciate nature more	Those who appreciate infrastructure more	
	(A)	(B)	
Non-acceptance of old trees (1W)	**A**		
Acceptance of old trees and biodiversity (2S)			
Approach to cutting trees (3CP)			

**Notes.**

Results are based on two-tailed tests, assuming equal variance, with a significance level of 0.05. For each significant pair, the smaller category appears below the category with the larger mean.

Depending on the variable–the letters are conventional. The letter always refers to the column heading, ie A–age 18-25. A–female, A–primary education.

## Discussion

The attractiveness of the urban green space planted with trees is influenced by many factors (Dwyer et al., 1992; Gołos, 2010; Summers et al., 2012; Suchocka, Jankowski & Błaszczyk, 2019; Suchocka, Błaszczyk & Kosno, 2019). It is worth emphasizing that few percent of city dwellers see negative aspects in trees (Suchocka et al., 2019; Wojnowska-Heciak et al., 2020), (Table 4). Those who visit municipal forest, assign little preference to young and usually dense stands (Pukkala, Kellomäki & Mustonen, 1988; Edwards et al., 2012) regardless of the tree species (birch, pine, or spruce). If young stands are more open, then their attractiveness increases because they are easier to penetrate (Gundersen et al., 2016). Generally, the preference increased with tree age, and more precisely, with tree size (Silvennoinen et al., 2001; Tyrväinen, Silvennoinen & Kolehmainen, 2003). According to Edwards and colleagues (Edwards et al., 2012), regardless of the European country considered, the more mature the forest, the better it is assessed (Hoen & Winther, 1993; Edwards et al., 2012). As the results show (Table 4), respondents declare a high degree of acceptance for hollow-bearing trees. This should convince urban forest managers to protect them and, together with the educated public, disregard negative pressure of tree arboriphobes (Suchocka, Jankowski & Błaszczyk, 2019) who do not accept trees in general. Users can name main types of habitats that determine biodiversity in some way, but they are frequently wrong (Kirkpatrick, Davison, Daniels, 2012; Bütler et al., 2013; Jonsson et al., 2016; Saine et al., 2018). For many people, dead decaying wood is an indicator of little diversity (dying), while such sites (niche) are extremely rich in species (Seibold et al., 2018). Respondents were unable to determine whether cutting branches improves the health of old trees but were not afraid of tree dying caused by presence of fruiting bodies of fungi (Table 3). We found an important relationship between the visiting frequency and the degree of acceptance of old hollow trees as wildlife habitats (Nilsson & Baranowski, 1997; Ranius & Nilsson, 1997; Wojnowska-Heciak, 2019; Mölder et al., 2020). Higher frequency of visits to urban forests reduced the willingness number of views related to the tree removal.

Similar conclusions were drawn by other researchers (Zelenski, Dopko & Capaldi, 2015; Rosa, Profice & Collado, 2018) whose findings indicate that contact with nature is positively associated with connectedness to nature and pro-environmental (environmentally sustainable) behaviours. What is interesting, young people appeared to be the group willing to make radical decisions about cutting down old and hollow trees. These results point out to two issues (and will perhaps contribute to further research). First of all, contact with nature is important for all decisions related to the management of green areas (and trees as such). Secondly, the importance of education should not be overlooked - the greater the awareness and knowledge of the environment, the greater the chance to take informed decisions that promote valuable elements of the environment. Perhaps greater practical involvement of students might be planned in schools’ curricula to increase young people’s enthusiasm for trees from direct experience. Our results are in line with those of other researchers, who indicate that less opportunities of contact with nature can result in an amplified feeling of human-nature dissociation (Chawla & Derr, 2012), which may result in less support for environmental causes (Soga & Gaston, 2016). Higher frequency of visits to urban forests enhances the level of interest and acceptance of old trees and helps get emotionally involved. Barro et al. (1997) found that emotional involvement of people and the importance of a tree increases with its importance in their memories and its special meaning to the people who know that tree (Barro et al., 1997). Potentially frequent contact builds up more memories and promotes the conservation of trees and associated biodiversity. Moreover, the findings confirm that emotional attachment plays an important role in motivating people to be concerned and share environmentally protective opinions (Vining, 1987; Vining & Schroeder, 1987; Vining, 1991).

The participation in the survey was voluntary and the questionnaire was distributed only through social media. Also, the respondents’ sample should go far beyond internet users with probable interest in municipal forest visits. However, despite this limitation of our study, research results show the role of respondents’ age in positive reception of old trees with and without hollows, as confirmed by other similar social studies (Kangur, 2015; Wojnowska-Heciak et al., 2020).

## Conclusions

Research on old trees should be extended to include detailed studies on cultural connections with old trees as well as on emotions evoked by various stages of tree maturity. Place attachment, spirituality and sacredness, cultural meaning, and all different types of dimensions of human interactions with old trees are a part of vanishing intangible heritage. Old trees are often a part of people’s memories from certain places or events. Experiencing a place covers not only physical characteristics of the surrounding but is also a psychological process worth studying in more detail. Emotions play a role in our everyday decisions and choices. It would therefore be interesting to investigate whether people’s feelings towards trees change over time at different tree life stages.

Equally important is the verification of the probability of the windfall of old hollow-bearing trees, in combination with healthy specimen. Tomography, resistograph testing and load tests would be decisive. In the context of climate change and the higher frequency of extreme phenomena, it is not obvious that the risk of damage caused by the old hollow-bearing trees is higher than the risk posed by a healthy tree. Quantitative research on the risk posed by trees with hollows in municipal forests and their contribution in preserving richness and stability of municipal forests would be extremely useful in the management process and as an educational approach. The data collected would provide a more complete picture of the biodiversity of municipal forests and help to fill the research and perception gap. For the urban ecosystem to be sustainable, include biodiversity, and reflect natural processes, the presence of mature and ancient hollow-bearing trees is indispensable.

## Supplemental Information

10.7717/peerj.12700/supp-1 Supplemental Information 1 Raw data analysis 1

10.7717/peerj.12700/supp-2 Supplemental Information 2 Raw data analysis 2

10.7717/peerj.12700/supp-3 Supplemental Information 3 Raw data analysis 3

10.7717/peerj.12700/supp-4 Supplemental Information 4 Mean values related to municipal forests visits and attitudes towards trees

10.7717/peerj.12700/supp-5 Supplemental Information 5 Analysis after 2 rounds of review

10.7717/peerj.12700/supp-6 Supplemental Information 6 Survey questions

## Additional Information and Declarations

### Data Availability

The following information was supplied regarding data availability: The data is available in the Supplemental Files.
