# Supplementary material for: Old trees are perceived as a valuable element of the municipal forest landscape
Source: PeerJ. 2022 Jan 12;10:e12700. doi: 10.7717/peerj.12700 (PMC8760855; doi:10.7717/peerj.12700)
Supplement: Supplemental Information 6 [file peerj-10-12700-s006.docx]

**Appendix 1. Survey questions**

1. Please select your gender
2. Female
3. Male
4. Other
5. Please select your age range

a)18-25 yrs

b)26-35yrs

c)36-45yrs

d)46-55yrs

e)56+

3. Please select your place of residence

1. Village
2. Town with less than 25 000 residents
3. Town 25 000 -100 000 residents
4. City with 101 000 - 500 000 residents
5. City with over 500 000 residents
6. Please select your education level:
7. Elementary
8. Vocational
9. Secondary
10. Students
11. Higher
12. How often do you visit municipal forest in Warsaw?
13. Everyday
14. Several times a week
15. Once a week
16. Once per several weeks
17. Once a month
18. Several times a year
19. Which of the forests do you visit? Choose five ansewrs.
20. Las Kabacki
21. Las Bielański
22. Las Młociński
23. Las Bemowski
24. Park Młociński
25. Las na Kole
26. Olszynka Grochowska
27. Las Bródno
28. Park Olszyna
29. Las Sobieskiego
30. Las Lindego
31. Kampinoski Park Narodowy
32. In the municipal forest in Warsaw you most appreciate? Choose five answers.
33. Connectness with nature
34. Beautiful landscapes
35. Comfortable hikig trails
36. Possibility of doing sports
37. Picnic sites
38. Playgrounds
39. Bike paths
40. Silence
41. Close distance
42. Calm and space
43. Possibility of walking with a dog
44. Other
45. Please rate the extent to which you agree/disagree with the following:

( 1-5 Likert scale

1 - I don't agree at all

2

3

4

5 - I totally agree)

1. Hollow trees pose a greater threat than trees without hollows.
2. Old trees increase the attractiveness of municipal forests.
3. Old trees are more valuable to the environment than younger trees.
4. It is better to replace an old tree with a younger one.
5. Fruiting bodies of fungi on the trunk or branches do not mean a death sentence for the tree on which they grow.
6. Hollow trees pose a greater threat than trees without hollows.
7. Decayed trees inside must be cut down immediately.
8. Dying trees should be cut down even if they are natural monuments.
9. Trees in municipal forests should undergo the same care as street trees.
10. Cutting branches improves the health of even old trees.
11. There are many methods to determine the actual health of very old trees.
12. The only way to ensure the safety of walkers is to cut down a threatening tree.

Thank you for submitting your answers.
